# A retrospective review of psychosocial correlates of chronic pain in cisgender, transgender, and gender-diverse youth receiving evaluation in interdisciplinary pediatric pain clinics

**DOI:** 10.1080/24740527.2025.2477466

**Published:** 2025-04-15

**Authors:** Crystal Tracy, Mary Lynch Milder, Lindsey Vater, Ann Lagges, Kathleen Lemanek, Sharon Wrona, Elaine Gilbert, Adam T. Hirsh, Megan M. Miller, Kelly Donahue, Morgan Streicher, Amy E. Williams

**Affiliations:** a Department of Psychology, Indiana State University, Terre Haute, Indiana, USA; b Great Lakes Neurobehavioral Center, Eagan, Minnesota, USA; c Department of Pediatrics, Nationwide Children’s Hospital, The Ohio State University College of Medicine, Columbus, Ohio, USA; d Department of Psychiatry, Indiana University School of Medicine, Indianapolis, Indiana, USA; e Department of Anesthesia and Pain Medicine, Nationwide Children’s Hospital, Columbus, Ohio, USA; f Department of Psychology, Indiana University–Indianapolis, Indianapolis, Indiana, USA; g Department of Pediatrics/Cincinnati Children’s Hospital Medical Center, Division of Behavioral Medicine and Clinical Psychology, University of Cincinnati School of Medicine, Cincinnati, Ohio, USA; h Department of Pediatrics, Indiana University School of Medicine, Indianapolis, Indiana, USA

**Keywords:** Gender-diverse youth, transgender youth, chronic pain, pediatric pain, psychosocial correlates

## Abstract

**Background:**

Individuals who experience social marginalization, such as transgender and gender-diverse (TGD) youth, have increased risk for poor health outcomes, including chronic pain. A better understanding of the impact of chronic pain in these populations would improve treatment and aid in reducing health care disparities. Our retrospective review of clinical data examined psychosocial correlates of pain in TGD and cisgender youth with chronic pain.

**Aims:**

The study aim was to explore differences in psychosocial variables between TGD and cisgender youth with chronic pain. In alignment with the minority stress model, we hypothesized worse pain and pain-related disability, poorer quality of life, and more internalizing symptoms in TGD patients. The secondary aim was to explore associations among psychosocial variables in TGD and cisgender youth.

**Methods:**

Data were collected from 140 youth (48 TGD, 92 cisgender) evaluated in pediatric pain clinics. Independent samples *t*-tests examined group differences in pain intensity, functional disability, quality of life, pain catastrophizing, and internalizing symptoms. Bivariate correlations were conducted for each group, and differences in the strength of correlations were evaluated using Fisher *r*-to-*z*. Institutional review board (IRB) approval was obtained for all study procedures at each participating institution prior to extraction of retrospective clinical data (Indiana 240 University IRB Protocol No. 12506, The Ohio State University College of Medicine IRB Protocol No. 16–00937). Informed consent was not required or obtained due to the retrospective nature of the study.

**Results:**

Cisgender patients reported worse pain intensity, whereas TGD patients reported lower quality of life and more internalizing symptoms. In the combined sample, pain intensity was correlated with worse functional disability, poorer quality of life, and more catastrophic thinking. No group differences in the strength of correlations were found.

**Conclusions:**

Results suggest that for TGD youth with chronic pain, internalizing symptoms and quality of life are important targets for treatment and improvement.

## Résumé

**Contexte :** Les personnes qui subissent une marginalisation sociale, telles que les personnes transgenres et de genres divers, présentent un risque accru de mauvaises issues de santé, notamment la douleur chronique. Une meilleure compréhension de l'effet de la douleur chronique au sein de ces populations permettrait d’améliorer les traitements et de contribuer à réduire des lisparités en matière de soins de santé. Notre revue rétrospective des données cliniques a examiné les corrélats psychosociaux de la douleur chez les jeunes transgenres et de genres divers et les jeunes cisgenres souffrant de douleur chronique.

**Objectifs :** L'objectif de l'étude était d'explorer les différences dans les variables psychosociales entre les jeunes transgenres et de genres divers et les jeunes cisgenres souffrant de douleur chronique. Conformément au modèle du stress minoritaire, nous avons formulé l'hypothèse d’une douleur plus grave et d'une invalidité liée à la douleur plus importante, d'une qualité de vie inférieure et de symptômes plus intériorisés chez les patients transgenres et de genres divers. L'objectif secondaire était d'explorer les associations entre les variables psychosociales chez les jeunes transgenres et de genres divers et les jeunes cisgenres.

**Méthodes :** Les données ont été recueillies auprés de 140 jeunes (48 transgenres et de genres divers, 92 cisgenres) évalués dans des cliniques de la douleur pédiatrique. Des tests t d'échantillons indépendants ont examiné les différences entre les groupes en matiére d'intensité de la douleur, d'invalidité fonctionnelle, de qualité de vie, de catastrophisation de la douleur et d'internalisation des symptômes. Des corrélations bivariées ont été menées pour chaque groupe, et les différences dans la force des corrélations ont été évaluées à l’aide de la transofrmation r-à-z de Fisher. L'approbation du comité d'examen institutionnel a été obtenue pour toutes les procédures de l'étude à chaque établissement participant avant l'extraction des données cliniques rétrospectives (Protocole IRB de l’Université de l’Indiana Indiana 240, Protocole IRB de la Faculté de médecine de l’Université d’État de l’Ohio No. 16–00937). Le consentement éclairé n'était pas requis en raison de la nature rétrospective de l'étude et n’a donc pas été obtenu.

**Résultats :** Les patients cisgenres ont signalé une intensité de la douleur plus faible, tandis que les patients de genres divers ont déclaré une qualité de vie inférieure et davantage de symptômes internalisés. Dans l'échantillon combiné, l'intensité de la douleur était corrélée à invalidité fonctionnelle plus grave, à une qualité de vie plus médiocre et à une pensée plus catastrophiste. Aucune différence entre les groupes n'a été trouvée quant à la force des corrélations.

**Conclusions :** Les résultats indiquent que chez les jeunes transgenres et de genres divers souffrant de douleur chronique, les symptômes internalisés et la qualité devie sont des cibles importantes pour le traitement et l'amélioration.

## Introduction

The International Association for the Study of Pain defines pain as an “unpleasant sensory and emotional experience associated with, or resembling that associated with, actual or potential tissue damage” and chronic pain as symptoms that persist or recur for 3+ months.^1^ Chronic pain in children and adolescents has risen in recent years, with as many as 46% of children and adolescents self-reporting pain occurring at least once a week for more than 3 months and 5% of that population indicating that their pain has led to functional impairments.^2^ Longitudinal studies indicate that about one-third of youth with chronic pain will continue to experience pain as adults.^3–5^ Chronic pain is a complex condition that is often associated with sleep disruption,^6–8^ functional impairment,^7,9–11^ chronic fatigue,^12^ school absences,^13^ symptoms of anxiety and depression,^14,15^ and social impairments.^16^ Pediatric chronic pain is estimated to cost the United States $19.5 billion annually.^17^ Individuals who experience social marginalization or stigma, such as transgender and gender-diverse youth, have increased risk for poor health outcomes including chronic pain.^18^ Further, chronic pain itself is a stigmatizing condition, and experience of pain-related stigma is associated with poorer health outcomes.^19^

The term gender-diverse is an *umbrella term* that encompasses all individuals who experience and express gender beyond the traditional binary, including those who identify as transgender, gender queer, agender, and non-binary. Though some transgender youth identify as gender-diverse, most transgender youth identify as either male or female but may not align with their sex assigned at birth. For simplicity in this article, transgender and gender-diverse will be abbreviated as TGD and will be used to encompass transgender, gender queer, agender, and non-binary youth. It is challenging to estimate the number of youth who identify as TGD; however, recent studies utilizing data from several national sources in the United States estimated that 1.2 million adults identify as non-binary^20^ and approximately 150,000 adolescents (13- to 17-year-olds) identify as transgender.^21,22^ A 2012 survey of 10,000 youth found that nearly 10% of 13- to 17-year-olds identified as TGD.^22^ A survey distributed in 2018 to nearly 5000 youth in urban school settings found a similar prevalence of 9.2% of youth identifying as TGD or not aligned with sex assigned at birth.^23^ Herman et al.^24^ reported that 1.4% of 13- to 17-year-olds identified as transgender. Canada’s 2021 Census data revealed that 1 in 300 people ages 15 and older in Canada identify as transgender or non-binary.^25^ Younger generations were more likely to identify as transgender or non-binary compared to older generations, with nearly two-thirds of this population being younger than 35.^26^ According to a 2023 report by the Human Rights Campaign Foundation,^27^ TGD youth face increased rates of harassment, peer rejection, bullying, and isolation and endorse elevated levels of stress, thereby increasing risk for poor health outcomes including pain. A survey of Swedish students found that compared to cisgender girls, TGD youth were more likely to report problems with pain.^28^ Another study found that among TGD youth, those who had experienced discrimination were twice as likely to have chronic pain.^29^

Pain treatment for individuals from minoritized groups remains complex, with numerous barriers. Limited research, inadequate assessment, and unsuccessful management have impeded tailoring care to suit the unique cultural needs of those individuals who have been historically marginalized.^30^ However, minority populations also depict incredible capacity in their strength, resilience, and advocacy to mobilize change.^31^ Rather than a singular focus on risk factors, it is essential that we also consider individual and community resilience factors as we research the interplay of biopsychosocial contributors to health.^18,31^

The minority stress model, which links the chronic stress response experienced by those in a marginalized group to negative physical and mental health outcomes, can be applied to TGD youth.^32^ There is evidence that suggests both distal stressors (e.g., mistreatment in the school setting, stigma and discrimination in health care settings, and bullying) and proximal stressors (e.g., fear of rejection or internalized negative thoughts about identity) contribute to adverse health outcomes in TGD youth.^33^ Misgendering of TGD persons can be a recurrent distal stressor, and verbal, physical, and sexual assault are common, with nearly half of TGD persons surveyed reporting that they had been victimized.^34^ Institutionalized social norms reflecting cultural ideologies of cisnormativity (i.e., “the assumption that it is ‘normal’ for one’s gender identity to reflect the physical sex assigned at birth in the expected way”^34^) and cisgenderism (i.e., prejudice that “denies, denigrates, or pathologizes self-identified gender identities that do not align with assigned gender at birth, as well as resulting behavior, expression, and community”^35^) contribute to the marginalization of, and prejudice toward, TGD persons.^34,36^ Exposure to institutionalized cisnormativity and cisgenderism can lead to the internalization of transphobia, leading to feelings of shame.^34^ Indeed, TGD youth are more likely to experience mental health symptoms compared to their cisgender peers.^28,37^ Adding the experience of chronic pain, a stigmatizing condition,^19,38^ can lead to even greater negative physical and emotional impacts of minority stress.^34,36^

TGD individuals experience significant health care disparities and barriers to accessing care. Such barriers include limited access to affirmative health care, poor insurance coverage, limitations of electronic medical records contributing to nonaffirmative care, socioeconomic barriers, and discrimination in health care settings.^39^ Gender-affirming health care is a patient-centered and trauma-informed approach that is sensitive to the needs and experiences of TGD patients.^40^ Nonaffirmative health care may include not using the identified name or pronouns, asking the patient to educate practitioners about TGD health care needs, or using outdated or offensive language.^40^ Nonaffirmative health care experiences are more common for non-binary or transmasculine adolescents compared to transfeminine adolescents, and past nonaffirmative experiences can contribute to health care avoidance.^40^ TGD adolescents report poorer health and lower health care utilization compared to their cisgender peers.^41,42^ Other research has identified higher mortality among transgender compared to cisgender adults.^43^ Consistent with experienced health care disparities, TGD individuals have not been suitably represented in pain research.^18,44,45^ The limited information available regarding pain treatment for TGD individuals suggests that best practice should promote an affirmative environment and consider interactions between mental health conditions, physical symptoms, pain treatment, community strengths, and gender-affirming medical care.^46,47^

A better understanding of the experience and impact of chronic pain in TGD youth would improve the treatment of pain and aid in reducing health care disparities. The current study was a retrospective review of clinical data to examine psychosocial correlates of pain in TGD and cisgender youth evaluated in interdisciplinary chronic pain clinics. In alignment with the minority stress model and the expected higher level of stress experienced by TGD youth, we hypothesized greater pain levels and pain-related disability, poorer quality of life, and greater internalizing symptoms in TGD patients compared to their cisgender peers. Given the novel nature of our research sample, our secondary aim was to explore potential differences in the relationships (i.e., strength of correlation) between variables within the TGD and cisgender patient groups.

## Methods

### Participants

This study includes retrospective clinical data collected from 140 adolescent patients seen for a new patient visit in two interdisciplinary pediatric pain management clinics in the Midwestern United States between August 2019 and January 2023. This study focused on youth given the potential for lifelong morbidity and economic cost of chronic pain that starts at a young age. During the time frame for the retrospective review, 48 adolescent patients identified as TGD (Site 1 = 33, Site 2 = 15) and were included in this study. A comparison group was selected consisting of 92 patients who identified as cisgender (Site 1 = 66, Site 2 = 26). For purposes of this study and due to sample size limitations, binary transgender youth and non-binary or gender queer (i.e., gender-diverse) youth were combined for comparison to cisgender youth. All patients who identified as TGD during the specified time frame were included in this study. TGD patients reported identities of trans man, gender queer, and non-binary.

Whenever possible two cisgender patients for each TGD patient were selected to create a comparison group. Cisgender patients were chosen who matched the TGD patients on the following criteria: age (within 1 year), primary pain diagnosis, date of appointment (within 3 months), and study site. This process was conducted individually for each TGD patient to provide the most representative comparison group. There were four TGD patients for whom there was only one appropriately matched cisgender patient. Race and ethnicity were not included in the matching profile due to limited racial and ethnic diversity in the sample.

Patients are referred to the participating interdisciplinary pain clinics for chronic or recurrent pain conditions often associated with primary pain disorders, chronic disease, injury, or surgery. New patient visits include comprehensive evaluations by a pain practitioner (medical doctor or advanced practice provider), pediatric psychologist, and physical therapist. Social workers are also included in the evaluation in one clinic and as needed at the other. As a part of standard of care in the participating clinics, patients and their parents/guardians complete questionnaires electronically prior to their first appointment. The present study includes data from these questionnaires and medical record review for demographic information, pain and psychiatric diagnoses, and information about sex, gender identity, and transition when relevant (including participation in gender-affirming medical interventions). After data extraction, all data were deidentified.

### Measures

Because this study was a retrospective analysis of clinical data from two independent pediatric pain clinics, measures utilized had some variability across sites. When the same measures were utilized by both sites, data were analyzed together. Where measures differed between sites (internalizing symptoms), separate analyses were done for each site/measure.

#### Demographic data

Medical record review was performed to gather patient demographic characteristics including age at date of appointment, pain diagnoses, mental health diagnoses, and gender identity. One of the participating pain clinics collected gender through a patient self-report form with a drop-down menu (“male,” “female,” “transgender,” “both,” “neutral,” “other”), and gender was confirmed in the medical record (from pain clinic documentation and/or demographics section). This form was sent to the patient’s parent/guardian, and it is not possible to determine whether the gender form was completed by the patient or parent. For the other pain clinic, gender was obtained from the medical record (pain clinic documentation and/or demographics section) and confirmed verbally with the patient and parent/guardian or self-reported verbally by the patient or caregiver before or during the initial appointment. Given that this is an adolescent population receiving care with a parent/guardian, the included TGD patients were open about their gender with the parent/guardian who was participating in their care in pain clinic. For patients identifying as TGD, medical notes were reviewed to code reported transition steps taken. Transition was coded as none or limited social transition (i.e., no one or very few people know their gender identity), social transition (i.e., patient reported living socially as their identified gender), and a combination of social, medical (e.g., nonsurgical interventions such as puberty blockers and/or hormone therapy), and/or legal (i.e., legally changing name and gender identity on formal documentation) transition. Additionally, data were gathered regarding whether TGD patients had received gender-affirming medical care, such as care from a dedicated gender clinic.

#### Pain intensity

Pain intensity ratings are self-reported data of a patient’s perceptions of the quality, extent, and/or degree of pain.^48,49^ Patients assessed their average pain intensity with a numerical rating scale (0–10, 0 = *no pain at all* to 10 = *most pain ever*).^50^ Pain intensity was assessed through the question, “In the past week, what is the average pain you have had?” at Site 1 and “What level of pain do you usually have?” at Site 2.

#### Functional Disability Index

The Functional Disability Index (FDI) is a 15-item self-report measure assessing physical functioning.^51^ Participants are asked to rate how much difficulty they experience completing various tasks at home, school, recreationally, and socially. Responses are made on a 0 to 4 Likert scale (0 = *no trouble* to 4 = *impossible*). Total scores range from 0 to 60; scores of 0 to 12 indicate no/minimal disability, 13 to 20 mild disability, 21 to 29 moderate disability, and ≥30 severe disability. The FDI is a well-validated and reliable measure in pediatric chronic pain.^52–54^ The FDI has acceptable to excellent internal consistency (α = 0.86–0.91) and good predictive validity.^51–53^ The FDI has shown high test–retest reliability at 2 weeks (child report, 0.74; parent report, 0.64) and moderate at 3 months (child report, 0.48; parent report, 0.39).^52^

#### Pain Catastrophizing Scale–Children

Pain catastrophizing is defined as “an exaggerated negative orientation towards actual or anticipated pain experiences.”^55^ The Pain Catastrophizing Scale–Children (PCS-C) is a 13-item scale (parent and self-report)^56^ assessing the extent of catastrophic beliefs about pain on a 5-point Likert scale (0 = *not at all* to 4 = *extremely*). Total score is a sum of all items and ranges from 0 to 52, with higher scores indicative of greater pain catastrophizing. Three subscales can be calculated: Rumination, Magnification, and Helplessness. The PCS-C has demonstrated good reliability and validity.^54,56,57^ The total PCS-C and subscales have acceptable to excellent internal consistency (ranging from α = 0.68 to 0.87).^56,57^

#### Pediatric Quality of Life Inventory

Functioning in emotional, social, and school domains was assessed with the Pediatric Quality of Life (PedsQL) Inventory–Generic Core Scales.^58,59^ This is a 23-item self-report measure with responses made on a 5-point Likert scale ranging from 0 = *never* to 4 = *almost always*. Items are reverse scored and transformed to a 0 to 100 scale. Higher scores reflect better quality of life or less impairment in the different domains of health. The PedsQL has been shown to have good reliability and validity in pediatric chronic health populations.^58,59^ Total HRQOL score was used in this study. The PedsQL has demonstrated excellent reliability for the total scale score (α = 0.89 child report; 0.92 parent report), for the physical health summary score (α = 0.88 child report; 0.88 parent report), and for the psychosocial health summary score (α = 0.83 child report; 0.86 parent report).^58,59^

#### Internalizing symptoms: Depression and anxiety

##### Site 1: Bath Adolescent Pain Questionnaire (BAPQ)–General Anxiety and Depression subscales

Depression and anxiety were assessed with the Depression and General Anxiety subscales of the BAPQ.^60^ These subscales are self-report measures asking patients to indicate how often in the last 2 weeks the statements have been true for them. The Depression subscale consists of six items (e.g., “I feel sad”), and the General Anxiety subscale consists of seven items (e.g., “I worry about the future”). Item responses are made on a 5-point Likert scale ranging from 0 = *never* to 4 = always. Subscale scores are a sum of item responses with ranges of 0 to 24 for depression and 0 to 28 for general anxiety, with higher scores reflecting worse symptoms. The BAPQ has been found to have good reliability and validity.^60^ The BAPQ subscales have demonstrated good internal consistency in a pain management sample (α= 0.80–0.85).^60^ Seven out of the eight BAPQ subscales have demonstrated good test–retest reliability for a 17-day interval in a pain management sample (*r*= 0.64–0.84).^60^

##### Site 2: Patient Health Questionnaire–9 Adolescent form (PHQ9-A) and Generalized Anxiety Disorder–7 (GAD-7)

The PHQ9-A is a validated nine-item self-report scale (4-point Likert scale) assessing symptoms of depression.^61,62^ Scores range from 0 to 27, with 0 to 4 indicating no or minimal depression, 5 to 9 mild depression, 10 to 14 moderate depression, 15 to 19 moderately severe depression, and 20 to 27 severe depression. Broadly, the PHQ-9 has demonstrated excellent internal reliability (α= 0.86–0.89), test–retest reliability, construct validity, and criterion validity.^61^ Additionally, the PHQ9-A has demonstrated good diagnostic validity, including satisfactory sensitivity, specificity, diagnostic agreement, and diagnostic accuracy, as compared to clinical interview.^62^

The GAD-7 is a seven-item screener for symptoms of anxiety over the past week.^63^ Each item is scored on a 0 to 3 scale and summed to create a total score that ranges from 0 to 21. Cut points of 5, 10, and 15 are used to identify mild, moderate, and severe levels of anxiety. The GAD-7 is an efficient screening tool and has been validated in adolescent^64^ and adult^63^ populations. The GAD-7 has demonstrated excellent internal consistency (α = 0.92) and test–retest reliability (intraclass correlation coefficient = 0.83).^63^

### Data analysis

Analyses were performed using SPSS v28 Data were assessed for missingness, normality, and outliers; >5% missingness was noted and determined to not be at random. Hot deck imputation^65^ was used with the following bins: TGD or cisgender, racial/ethnic majority or minority, site, and diagnosis of fibromyalgia (yes or no). This diagnosis was chosen as a bin given the high frequency in the sample. Following imputation, <1% of missingness was noted and determined to be at random. *t*-Tests were used to compare the original variables to the imputed variables. No significant differences on any of the measures were noted, indicating that the imputation method appropriately enhanced the data set.

Descriptive statistics, including means and standard deviations for continuous variables and frequencies for categorical variables, are presented in Table 1. Due to small samples sizes for racial and ethnic minority groups, categories were combined into racial and ethnic majority (i.e., White, non-Hispanic) and racial and/or ethnic minority.

**Table 1.** Sample demographic characteristics.

	TGD (*n* = 48)		Cisgender (*n* = 92)		Full sample (*N* = 140)	
	*n*	%	*n*	%	*N*	%
Gender identity						
Female	0	0	77	83.7	77	55
Male	0	0	15	16.3	15	10.7
Trans man (AFAB)	29	60.4	0	0	29	20.7
Trans woman (AMAB)	0	0	0	0	0	0
Non-binary or gender queer	19	39.6	0	0	19	13.6
Race and ethnicity						
White, non-Hispanic	42	87.5	76	82.6	119	85
Racial and/or ethnic minority	6	12.5	16	17.4	21	15
Pain condition						
Fibromyalgia, AMPS, or widespread chronic pain	32	66.7	60	65.2	92	65.7
Hypermobility syndrome or Ehlers-Danlos syndrome	8	16.7	13	14.1	21	15
Chronic back pain	6	12.5	16	17.4	22	15.7
Joint pain	6	12.5	12	13.0	18	12.9
Headaches, migraines	10	20.8	24	26.1	34	24.3
Abdominal pain	6	12.5	12	13.0	18	12.9
Arthritis	2	4.2	2	2.2	4	2.9
Neuropathy	2	4.2	1	1.1	3	2.1
Extremity pain	5	10.4	5	5.4	10	7.1
Neck pain	0	0	2	2.2	2	1.4
Shoulder pain	0	0	2	2.2	2	1.4
Other specific conditions	2	4.2	6	6.5	8	5.7
Psychiatric diagnoses						
Anxiety disorder	46	98.8	59	64.1	105	75
Psychological factors affecting medical condition	19	39.6	43	46.7	62	44.3
Depressive disorder	38	79.2	38	41.3	76	54.3
Trauma- and stressor-related disorder	9	18.8	12	13.0	21	15
ADHD	11	22.9	5	5.4	16	11.4
Insomnia	3	6.3	8	8.7	11	7.9
FNSD, conversion, somatic disorder	0	0	3	3.3	3	2.1
Other disorder	7	14.6	6	6.5	13	9.3
No psychiatric diagnosis	0	0	8	8.7	8	5.7
	*M*	SD	*M*	SD	*M*	SD
Age	15.98	1.24	16.00	1.28	15.99	1.26

Due to some patients having multiple pain and psychiatric diagnoses, sum of percentages does not equal 100% for those categories. TGD = transgender and gender-diverse. AFAB = assigned female at birth. AMAB = assigned male at birth. AMPS = amplified musculoskeletal pain syndrome. EDS = Ehlers’s Danlos Syndrome. ADHD = Attention Deficient Hyperactivity Disorder. FNSD = Functional Neurological Symptom Disorder. Percentages represent the % of the sample in the corresponding column.

Independent samples *t*-tests examined group differences (TGD compared to cisgender) in pain intensity (0–10 NRS), functional disability (FDI), quality of life (PedsQL), pain catastrophizing (PCS), and internalizing symptoms. Bivariate correlations between variables were assessed using Pearson correlation coefficients. Differences in the strength of the correlations between cisgender and TGD patients were evaluated using Fisher’s *r*-to-*z* transformation.^65,66^

## Results

### Descriptive statistics

Patients were adolescents (*M* =15.99 years; SD 1.26; range, 12–18) primarily of White, non-Hispanic race and ethnicity (*N* =119, 85%). Of note, biological sex categories (i.e., male and female) were used to describe cisgender youth because these were the terms used in the questionnaires used for the present study. Participant gender identity was reported as cisgender female (*N* =77, 55%), cisgender male (*N* =15, 11%), transgender boys/men (*N* =29, 21%), and non-binary or gender queer (*N* =19, 14%). One-third of TGD youth were on puberty blockers and/or hormone therapy (*N* =16). Regarding transition steps taken, 14 reported none or limited social transition, 27 reported social transition only, and 7 reported a combination of social, medical (nonsurgical), and/or legal transition.

The most common pain diagnosis for the entire patient sample was fibromyalgia or amplified pain syndrome (*N* =92, 66%), followed by headaches and migraines (*N* = 34, 24%) and chronic back pain (*N* = 22, 16%). Of note, amplified pain syndrome is an idiopathic musculoskeletal pain diagnosis, of which fibromyalgia is a diagnostic subset, involving either central or peripheral sensitization of pain.^67^ For cisgender participants, the most reported pain diagnosis was fibromyalgia or amplified pain syndrome (*N* = 60, 65%), followed by headaches and migraines (*N* = 24, 26%) and chronic back pain (*N* = 16, 17%). For TGD participants, the most reported pain diagnosis was fibromyalgia or amplified pain syndrome (*N* = 32, 67%), followed by headaches and migraines (*N* = 10, 21%) and Ehlers-Danlos syndrome or hypermobility (*N* = 8, 17%). Approximately 51% of the entire sample reported having at least two pain diagnoses, and 16% reported having three pain diagnoses.

The most common mental health diagnoses reported by participants were anxiety (total sample *N* = 105, 75%; cisgender *N* = 59, 64%; TGD *N* = 46, 96%), depression (total sample *N* = 76, 54%; cisgender *N* = 38, 41%; TGD *N* = 38, 79%), and psychological factors affecting medical conditions (total sample *N* = 62, 44%; cisgender *N* = 43, 47%; TGD *N* = 19, 40%). Approximately 87% of the entire sample reported having at least one mental health diagnosis, 74% reported at least two, 44% reported at least three, 14% reported at least four, and 6% reported five mental health diagnoses.

### Group differences

Results of independent samples *t*-tests are included in Table 2. There were no significant differences in the number of pain diagnoses reported by cisgender and TGD youth. Cisgender patients reported greater pain intensity (*t*_138_ = 3.12, *P* < 0.01, *d* = 0.55). TGD patients reported lower quality of life (*t*_136_ = 2.29, *P* < 0.05, *d* = 0.41). TGD patients also endorsed more symptoms of anxiety (BAPQ *t*_77_ = −2.05, *P* < 0.05, *d* = −0.41; GAD7 *t*_39_ = −1.34, *P* = 0.09, d = −0.44) and depression (BAPQ *t*_97_ = −2.57, *P* < 0.01, *d* = −0.55; PHQ9 *t*_39_ = −1.62, *P* = 0.057, *d* = −0.52). There were no significant differences on functional disability or pain catastrophizing.

**Table 2.** Independent samples *t*-test.

	Cisgender *M* (SD)	TGD *M* (SD)	*t*	*P*	Cohen’s *d*
Average pain score (past week) (*N* = 92; *N* = 48)	5.68 (1.96)	4.56 (2.14)	3.12	<0.01	0.56
Functional Disability Inventory (*N* = 92; *N* = 48)	26.86 (11.35)	29.35 (10.84)	−1.25	0.11	0.22
PedsQL total score (*N* = 90; *N* = 48)	44.98 (15.93)	38.45 (16.06)	2.29	<0.05	0.41
Pain Catastrophizing (*N* = 92; *N* = 48)	31.48 (14.04)	34.25 (13.14)	−1.13	0.13	0.20
BAPQ Depression (*N* = 66; *N* = 33)	12.26 (5.33)	15 (4.28)	−2.57	<0.01	0.55
BAPQ Anxiety (*N* = 66; *N* = 32)	14.64 (6.82)	17.22 (5.31)	−2.05	<0.05	0.41
PHQ-9 (*N* = 26; *N* = 15)	12.58 (6.41)	15.73 (5.28)	−1.62	0.06	0.52
GAD-7 (*N* = 26; *N* = 15)	10.62 (5.28)	13 (5.66)	−1.36	0.09	0.44
Number of pain diagnoses	1.68 (0.74)	1.65 (0.76)	0.29	0.77	0.052

TGD = Transgender and Gender-Diverse. PedsQL = Pediatric Quality of Life Scale. BAPQ = Bath Adolescent Pain Questionnaire. PHQ9 = Patient Health Questionnaire – 9, GAD7 = Generalized Anxiety Disorder – 7

### Correlations

In the full sample (Table 3), higher pain intensity was correlated with greater functional disability (*r* =0.34, *P* < .001), worse quality of life (*r* = −0.23, *P* < 0.001), and more catastrophic thinking (*r* = 0.43, *P* < 0.001). No significant differences in the strength of correlations were found between cisgender and TGD patients (Tables 4 and 5).

**Table 3.** Means, standard deviations, and bivariate correlations for psychosocial variables in cisgender patients with chronic pain.

Variable	1	2	3	4	5	6	7
1. Average pain score	–						
2. Functional Disability Index	0.35**	–					
3. PedsQL total score	−0.24*	−0.54**	–				
4. Pain Catastrophizing	0.45**	0.48**	−0.35**	–			
5. BAPQ Depression	0.16	0.54**	−0.63**	0.57**	–		
6. BAPQ Anxiety	0.08	0.49**	−0.57**	0.63**	0.81**	–	
7. GAD-7	0.33	0.58**	−0.35	0.36	–	–	–
8. PHQ-9	0.24	0.65**	−0.47*	0.40*	–	–	0.76**

*p < 0.05; **p < 0.01. PedsQL = Pediatric Quality of Life Scale. BAPQ = Bath Adolescent Pain Questionnaire. PHQ9 = Patient Health Questionnaire-9, GAD7 = Generalized Anxiety Disorder-7.

**Table 4.** Means, standard deviations, and bivariate correlations for psychosocial variables for transgender and gender-diverse patients with chronic pain.

Variable	1	2	3	4	5	6	7
1. Average pain score	–						
2. Functional Disability Index	0.42**	–					
3. PedsQL total score	−0.38**	−0.59**	–				
4. Pain Catastrophizing	0.51**	0.46**	−0.59**	–			
5. BAPQ Depression	0.42*	0.58**	−0.57**	0.44*	–		
6. BAPQ Anxiety	0.35	0.54**	−0.65**	0.57**	0.81**	–	
7. GAD-7	−0.24	−0.09	−0.31	0.61*	–	–	–
8. PHQ-9	−0.12	−0.02	−0.62*	0.35	–	–	0.66**

*p < 0.05; **p < 0.01. PedsQL = Pediatric Quality of Life Scale. BAPQ = Bath Adolescent Pain Questionnaire. PHQ9 = Patient Health Questionnaire-9, GAD7 = Generalized Anxiety Disorder-7.

**Table 5.** Means, standard deviations, and bivariate correlations for psychosocial variables for full sample.

Variable	1	2	3	4	5	6	7
1. Average pain score	–						
2. Functional Disability Index	0.34**	–					
3. PedsQL total score	−0.23**	−0.56**	–				
4. Pain Catastrophizing	0.43**	0.47**	−0.44**	–			
5. BAPQ Depression	0.17	0.55**	−0.62**	0.55**	–		
6. BAPQ Anxiety	0.11	0.51**	−0.59**	0.62**	0.81**	–	
7. GAD-7	0.04	0.40*	−0.37*	0.39*	–	–	–
8. PHQ-9	0.02	0.51**	−0.54**	0.31	–	–	0.73**

*p < 0.05; **p < 0.01. PedsQL = Pediatric Quality of Life Scale. BAPQ = Bath Adolescent Pain Questionnaire. PHQ9 = Patient Health Questionnaire-9, GAD7 = Generalized Anxiety Disorder-7.

## Discussion

There remains a dearth of research on chronic pain in TGD youth.^68^ The minority stress model suggests that TGD youth experience stigma, prejudice, and discrimination that creates hostile stressors unique to their identity.^69^ Likely due to expected higher levels of acute and chronic stress, TGD youth report experiencing worse physical and mental health outcomes than their cisgender counterparts.^28,70^ The current study evaluated psychosocial correlates of chronic pain among 48 TGD youth and matched cisgender peers who were evaluated in one of two included outpatient integrated pediatric pain clinics in the Midwestern United States. Results of the study suggest that TGD youth seeking treatment at pediatric pain clinics report experiencing lower quality of life and more symptoms of anxiety and depression than their cisgender peers. In other ways, TGD and cisgender youth were more similar than different. For both groups, higher pain intensity was associated with greater functional disability, worse quality of life, and more worries about pain (pain catastrophizing scale). Further, there were no group differences in the strengths of the correlations among these variables.

Our findings are consistent with previous research outlining the higher prevalence of mental health symptoms in TGD youth as compared to cisgender youth,^28,37,71^ including among youth with chronic pain.^70^ A recent study by Scheurich et al.^70^ evaluated psychosocial variables among 30 TGD adolescents and 30 matched cisgender youth who participated in an intensive (40 h per week for 3 weeks) interdisciplinary pain treatment program for treatment of chronic pain and/or orthostatic intolerance. Similar to our results, this study found that prior to participating in intensive treatment, TGD youth had more depression and anxiety compared to matched cisgender peers.^70^ Also similar to our results, they did not find any difference between groups on pain catastrophizing.^70^ However, in contrast to our findings, Scheurich et al.^70^ found no difference in pain intensity between groups and greater functional disability in TGD versus cisgender youth.

These data indicate that TGD youth with chronic pain experience lower quality of life and more symptoms of anxiety and depression. TGD youth are at increased risk for experiencing stressful social environments that predispose them to and perpetuate mental health problems.^69^ The challenges faced by TGD youth due to cisgenderism, transphobia, and victimization may be compounded by the physical and mental stressors associated with chronic pain.^19,38^ The minority stress model proposes that the stressors experienced by TGD youth associated with social marginalization due to a culture of cisnormativity and due to the experience of chronic pain would put TGD youth at an increased risk for greater disability, more mental health symptoms, greater pain catastrophizing, and higher pain intensity. However, our results did not fully support this hypothesis. We found no significant differences in functional disability or pain catastrophizing between TGD and cisgender youth. Moreover, cisgender youth reported greater pain intensity than their TGD peers, which was also counter to the hypotheses. Other findings did support our hypothesis; TGD youth with chronic pain reported worse quality of life and greater mental health symptoms than their cisgender peers.

Although the present study does not enable verification, we propose several possible explanations for why the study hypotheses were not fully supported. One explanation for these findings may be that there was a greater focus on psychological distress, compared to physical health concerns, in TGD youth’s self-reports. Experiences of discrimination and marginalization associated with gender identity may contribute to increased psychological distress. Another explanation may be that TGD youth are more vulnerable to mental health comorbidities because of the additional stressors associated with the intersectionality of chronic pain stigma and the impact of cisgenderism, marginalization, and discrimination on TGD youth. This is consistent with Scheurich et al.’s^70^ findings indicating that TGD youth attending an intensive interdisciplinary pain treatment program had more emotional distress compared to cisgender peers in the program. Cisgender youth reported greater pain intensity than TGD peers but had lower mental health symptoms and equivalent disability and pain catastrophizing to TGD peers. Consistent with the minority stress model, one potential explanation for this finding is that TGD youth may be more susceptible to negative physical and mental health outcomes in the context of lower levels of chronic pain intensity. Scheurich et al.^70^ did find significantly more functional disability among TGD youth with chronic pain compared to their cisgender peers; however, there was no difference in pain intensity across TGD and cisgender youth in their study. Given our finding that pain intensity was correlated with functional disability in TGD and cisgender youth, it is likely that TGD youth would have had greater functional disability if pain intensity was not significantly lower than that of their cisgender peers. As such, future work should evaluate how pain intensity impacts functional disability outcomes in TGD and cisgender youth.

Another possible explanation for the absence of a difference on functional disability is that a selection bias may have led to the study only including participants with similar levels of disability. This study included patients who were evaluated in an interdisciplinary chronic pain clinic. Functional disability may be a key factor for providers to use to determine need for referral to a pain clinic, and thus only youth with similar levels of disability are referred. Future work should further evaluate functional disability in a broader sample of TGD and cisgender youth with chronic pain who have not been referred to dedicated pain clinics. The present data do not enable us to evaluate which of these hypotheses best explains the findings, and future work is needed to elucidate causal factors.

Based on the current results, TGD youth with chronic pain may be at risk for poorer mental health and quality of life outcomes with similar levels of pain intensity as cisgender peers. However, it is also possible that TGD youth are less likely to disclose physical symptoms to health care providers due to previous experiences with stigma, discrimination, invalidation, or minimization of symptoms from health care providers. One study found that 28% of TGD individuals surveyed reported postponing or avoiding needed medical treatment, and 33% reported that they delayed or did not go to get the care they needed because of disrespect and discrimination from medical providers.^72^ Additionally, it would be beneficial to replicate this study with enough male cisgender patients (the present sample is predominantly female) to allow comparison between TGD and cisgender youth matched for identified gender. Sex differences in pain are well documented, with females reporting more pain, and prior work has indicated that gender identity may play a greater role than biological sex.^73^ Therefore, gender differences in reporting of pain may have contributed to group differences in the present study. A better understanding of causal factors leading to lower quality of life and increased mental health symptoms for TGD youth with chronic pain is needed to inform interventions to reduce negative outcomes and improve coping.

It is important to note that the PCS was used in this study to assess pain-related worries. The pain research community has recently been engaged in discussion about stigma associated with the term “pain catastrophizing.”^74,75^ Given this ongoing debate about use of the term pain catastrophizing to describe pain-related worries, future work should be careful to consider implications of how the PCS is interpreted. Care should be taken to avoid the implication that pain catastrophizing equates to exaggeration of pain or implies a psychogenic etiology for pain. This may be of particular concern for patients who may experience stigma for multiple reasons, such as TGD adolescents who also have chronic pain.

### Implications

A recent editorial has called for efforts to improve inclusion and diversity in pain research.^76^ Research on the experience and effects of chronic pain has primarily been conducted with presumed cisgender patients, leaving a significant gap in our understanding of chronic pain in TGD youth. The current study suggests that quality of life and internalizing symptoms are especially important treatment targets for TGD youth with chronic pain.

One pathway to improving quality of life for TGD youth with chronic pain may be ensuring that interactions with patients are gender affirmative. TGD youth often face stigma and discrimination in and out of the health care system. Gender-affirmative interactions include use of identified names and pronouns, coordinating care to support their gender identity, and being an ally and advocate for the patient. When discussing their health care, TGD youth report that it is important for providers to ask about their gender and pronouns and for providers to have received education on TGD health.^77^ These actions can help improve the patient’s health care, quality of life, and mental health symptoms,^78^ which may contribute to improvements in their chronic pain. Based on current findings, the minority stress model provides a useful framework to examine health disparities among TGD youth experiencing chronic pain.^44,69^

### Limitations and future directions

To our knowledge, this is one of the first studies, along with work by Scheurich et al.,^70^ to examine and compare the psychosocial experiences of TGD and cisgender youth with chronic pain. These findings should be considered in light of important limitations. The TGD youth included were open to self-reporting their gender to providers in the presence of their participating parent/guardian, which does not allow analysis of those who did not yet feel comfortable disclosing their gender. Future studies may benefit from anonymous surveys of adolescents to also capture the experience of those who have not yet disclosed their gender identity to family. Information included in this article regarding transition is limited by what was available in the medical record and identified by one researcher who completed medical record review. Thus, it is possible that this information is not complete. It would be of benefit to gather additional data on the extent of social and medical transition including barriers and facilitators to desired steps in transition. We did not formally screen for mental health concerns other than anxiety and depression. TGD youth face unique stressors related to their identity that may lead to a variety of mental health concerns. As such, future research should examine other mental health outcomes, such as trauma, to determine their prevalence and impact among TGD youth with chronic pain.

Additionally, the current study was underpowered to examine differences within TGD youth based on age, race, ethnicity, other intersectional identities, or steps to transition. Due to sample size constraints, all TGD youth were grouped together, regardless of identity as transgender, non-binary, or gender queer and regardless of racial and ethnic background or steps to transition. TGD youth from minority backgrounds and at different steps in their transition process may experience unique physical and mental health outcomes. Additionally, only two study sites were included, both of which are located in the Midwestern United States. Cultural differences related to geographical regions may impact patient’s comfort disclosing gender identity. Future research with larger sample sizes, across broader geographical areas, is needed to better understand within-group differences and intersectional nuances among TGD youth with chronic pain. All patients were presenting to an interdisciplinary pediatric pain clinic for evaluation. Broadening recruitment strategies outside of pain clinics would provide more generalizable data.

Given that this was a retrospective review of clinical data, there were limitations in what data were available for analysis. We did not have access to individual item-level data for the measures utilized, which prevented calculation of psychometrics and internal validity among the present sample. Further, the measures have not been formally validated in a TGD population, and future work should seek to verify scale validity in this population. Finally, given that we included patients from two separate integrated pain clinics, there were some differences in measures utilized, as noted in the Methods section. In some cases where measures were different, this prevented combining data across sites for analysis. For pain intensity ratings, both sites used a 0 to 10 NRS scale and data were combined for analysis. However, it is important to note that the prompts for completing pain intensity ratings were different across sites, with one site asking about “average pain” in the past week and the other asking about “usual pain.” Because both were assessing a general average pain, we chose to combine the measures.

### Conclusions

Practical implementation of gender-affirmative care includes providing validation and support of the identities of the patients; assessing the impact and interplay of stigma, prejudice, and discrimination; being an ally and advocate for patients; and engaging in evidence-based practice from a biopsychosocial framework. The current study suggests that TGD youth with chronic pain may especially benefit from interventions focused on quality of life and internalizing symptoms. Pediatric pain clinics may choose to adopt equity-oriented health care approaches that integrate quality of life and internalizing interventions with trauma- and violence-informed care, culturally safe care, and harm reduction^45^ to specifically target the needs of TGD youth with chronic pain. Pediatric psychologists, pediatricians, pain treatment providers, and associated health care team members are in a unique position to both advance the research on chronic pain in TGD youth and to ensure the implementation of evidence-based, gender-affirmative care for TGD youth with chronic pain.

## Supplementary Material

TrackedFeb25_CanJPain_ChronicPainTGDYouth.docx
